# Supplementary material for: Magnitude of job satisfaction and intention to leave their present job among nurses in selected federal hospitals in Addis Ababa, Ethiopia
Source: PLoS One. 2022 Jun 7;17(6):e0269540. doi: 10.1371/journal.pone.0269540 (PMC9173620; doi:10.1371/journal.pone.0269540)
Supplement: Attachment — Submitted filename: Response to Reviewers.docx [file pone.0269540.s002.docx]

**Dear** Prof. Petri Böckerman**,**

Thank you for giving us the opportunity to submit a revised draft of our manuscript titled “Magnitude of job satisfaction and intention to leave present job among nurses in selected Federal Hospitals in Addis Ababa, Ethiopia” to *Plos One journal*. We appreciate you and the reviewers for your precious time in reviewing our paper and providing valuable suggestions. It was your valuable and insightful comments that led to possible improvements in the current version. The authors have carefully considered the comments and tried our best to address every one of them. We hope the manuscript after careful revisions meet your high standards. The authors welcome further constructive comments if any.

Below we have provided the point-by-point responses to editor and reviewers comments. All modifications in the manuscript have been highlighted in red.

1. **Comments from Editor :**

**Comment 1:** There is a substantial body of literature on quit intentions that is not covered in the manuscript (see https://doi.org/10.1111/j.1468-232X.2008.00546.x). This should be stated in the paper

**Response:** Thank you for recommending this valuable scientific paper to include in our manuscript. We have included in introduction part as reference number 24.

**Comment 2:** If applicable, we recommend that you deposit your laboratory protocols in [protocols.io](http://protocols.io/) to enhance the reproducibility of your results.

**Response:** Thank you for your suggestion. We have deposited our source data in protocols.io. https://www.protocols.io/file-manager/343F4601834C11ECA0DE0A58A9FEAC02 (dx.doi.org/10.17504/protocols.io.b5b6q2re)

2. **Comments from reviewer 1:**

**Comment 1:** There is substantial literature published on the area present by the manuscript.

**Response:** Thank you for your valuable comment. We agree that there are some literatures done in this area previously. However, the literatures published are not enough to show this sound problem in the country, specifically study area. Addis Ababa is a place where huge and complex health problems are screened and treated. This needs an adequate number of well experienced health care professionals to appropriately respond those public health problems. As such, hospitals need to retain experienced health care professionals including nurses. To make this practical, additional evidences are needed to know the overall prevalence of the problem (Pooled prevalence), and suggest to policy makers for interventions.

**Comment 2**: There is very little that the findings of the study at to the existing database.

**Response:** Thank you again for the comment: We disagree with the comment. We belief that the finding of this study will provided additional input to the existing literature to show the overall magnitude of the problem.

**Comment 3:** No cause-effect relation can be established due to study design limitations

**Response:** Thank you again for additional input: We agreed with the comment. Due to design limitation, the study do not established cause-effect relationship; however, we recommended longitudinal and interventional study for future researches to fill this drawback.

3. **Comment from reviewer 2:**

**Comment 1:** This is an interesting paper depicting the loss of health care workers and the reasons for mass resignation. It is a relevant topic which must be brought to the attention of management and likeminded individuals. While the technicalities of the paper were simple, the underlying message is clear.

**Response:** Thank you for the interesting understanding and explanation of the problem.

**Comment 2:** For the paper to be accepted, a native English speaker should review the paper to improve the language.

**Response:** We have used experienced professionals to edit the paper from both English language and content point of view.

**Comment 3:** I also highly recommend a thorough formatting of the paper for publication in the journal.

**Response:** We have tried to format the paper according to the journal formatting style.

Comment 4: There is also a missed opportunity to discuss further the sort of interventions that might allow for prevention of loss of nurses.

**Response**: Thank you for interesting point. We have tried to discuss possible sort of interventions in both discussion and recommendation parts.

4. **Additional clarification to the journal**

**Comment 1:** Please ensure that your manuscript meets PLOS ONE's style requirements, including those for file naming

**Response:** Thank you for your suggestion. We have tried all our best in revising the manuscript as per the journal format.

**Comment 2:** We suggest you thoroughly copyedit your manuscript for language usage, spelling, and grammar

**Response:** Thank you for your suggestion. We have tried to correct all language usage, spelling, and grammar errors. The manuscript was edited by professional who have sophisticated experience in document editing and publication in different journal including Plos journals. In addition, we have used AJE free trial version to make our paper more editorially fit to the journal requirement.

**Comment 3:** We note that you have indicated that data from this study are available upon request. PLOS only allows data to be available upon request if there are legal or ethical restrictions on sharing data publicly

**Response:** Thank you for the comment. To adhere to the journal data policy we agreed to provide our source data. We have uploaded as supporting information. Additionally, we have deposited our source data in protocols.io. (dx.doi.org/10.17504/protocols.io.b5b6q2re)

**Comment 4:** PLOS requires an ORCID iD for the corresponding author in Editorial Manager on papers submitted after December 6th, 2016.

**Response.** Thank you for your comment. Both corresponding authors provided their ORCID iD.

**Comment 5:** Your ethics statement should only appear in the Methods section of your manuscript.

**Response:** Thank you for your point. We have included ethical statement in the method part.

**Comment 6:** We note you have included a table to which you do not refer in the text of your manuscript. Please ensure that you refer to Table 3 in your text;

**Responses:** Thank you for your point. Table 3 was cited at the end of the paragraph found immediately before table 3.

**Comment 7:** We noticed you have some minor occurrence of overlapping text with the following previous publication(s), which needs to be addressed

**Responses:** Thank you for the comment. We have corrected and tried to remove all similarly issues with the previous papers. We cited all sources we have used.
