# Supplementary material for: Magnitude of job satisfaction and intention to leave their present job among nurses in selected federal hospitals in Addis Ababa, Ethiopia
Source: PLoS One. 2022 Jun 7;17(6):e0269540. doi: 10.1371/journal.pone.0269540 (PMC9173620; doi:10.1371/journal.pone.0269540)
Supplement: Attachment — Submitted filename: Response to Reviewers.docx [file pone.0269540.s003.docx]

**Dear** Prof. Petri Böckerman**,**

Thank you for giving us another opportunity to submit a revised draft of our manuscript titled “Magnitude of job satisfaction and intention to leave present job among nurses in selected Federal Hospitals in Addis Ababa, Ethiopia” to *Plos One journal*. We appreciate you and the reviewers for your precious time in reviewing our paper and providing valuable suggestions. It was your valuable and insightful comments that led to possible improvements in the current version. The authors have carefully considered the comments and tried our best to address all the comments and suggestions. We hope the manuscript after careful revisions meet your high standards.

Below we have provided the point-by-point responses to reviewer #2 comments. All modifications in the manuscript have been highlighted in red.

**Comment from reviewer #2**

- While the authors have tried to address most of the queries from the editor and the reviewers, the quality of language is still poor in the manuscript. Authors are advised to check on acronyms, capitalization, tense and sentence structure to be published in a international journal. Again I advise a professional English service for this.

**Response to reviewer #2**

- As the comments of reviewer #2, we tried to improve the quality of the manuscript’s language. We check all the acronyms, capitalization, tense and sentence structure as the quality of publication permits. Furthermore, we tried to get professional English language support from American Journal Experts.
